# Integrative microRNA and gene expression analysis identifies new epigenetically regulated microRNAs mediating taxane resistance in ovarian cancer

**DOI:** 10.1038/s41598-020-78596-5

**Published:** 2021-01-12

**Authors:** Mohamed K. Hassan, Amr A. Waly, Waheba Elsayed, Sarah Keshk, Walaa Ramadan Allam, Sherif F. El-khamisy

**Affiliations:** 1 Center for Genomics, Helmy Institute for Medical Sciences, Zewail City of Science and Technology, Giza, Egypt; 2 Bitechnology Program, Zoology Department, Faculty of Science, Port Said University, Port Said, Egypt; 3 Department of Molecular Biology and Biotechnology, Krebs Institute, University of Sheffield, Sheffield, UK; 4 Institute of Cancer Therapeutics, University of Bradford, Bradford, BD7 1DP UK

**Keywords:** Cancer epigenetics, Ovarian cancer

## Abstract

Taxane is a family of front-line chemotherapeutic agents against ovarian cancer (OC). The therapeutic efficacy is frequently counteracted by the development of chemoresistance, leading to high rates of relapse in OC patients. The role(s) of microRNAs (miRNAs) in cancer chemoresistance had been supported by many evidences Epigenetic regulation by miRNAs has been reported to influence cancer development and response to therapeutics, however, their role in OC resistance to paclitaxel (PTX) is unclear. Here, we conducted miRNA profiling in the responsive and PTX-resistant OC cell lines before and after treatment with epigenetic modulators. We reveal 157 miRNAs to be downregulated in the PTX-resistant cells compared to parental controls. The expression of five miRNAs (miRNA-7-5p, -204-3p, -501-5p, -3652 and -4286) was restored after epigenetic modulation, which was further confirmed by qPCR. In silico analysis of the signaling pathways targeted by the selected miRNAs identified the PI3K-AKT pathway as the primary target. Subsequent cDNA array analysis confirmed multiple PI3K-AKT pathway members such as AKT2, PIK3R3, CDKN1A, CCND2 and FGF2 to be upregulated in PTX-resistant cells. STRING analysis showed the deregulated genes in PTX-resistant cells to be primarily involved in cell cycle progression and survival. Thus, high throughput miRNA and cDNA profiling coupled with pathway analysis and data mining provide evidence for epigenetically regulated miRNAs-induced modulation of signaling pathways in PTX resistant OC cells. It paves the way to more in-depth mechanistic studies and new therapeutic strategies to combat chemoresistance.

## Introduction

Ovarian carcinomas (OCs) have the highest mortality rates among gynaecologic cancers. This is attributed to the fact that OCs are asymptomatic in the early tumorogenesis stage making it the fifth leading cause of cancer death in women with an overall survival rate of only ~ 40%. Over 220,000 women are diagnosed with OC every year worldwide and around 14,000 die annually in the U.S. alone^1,2^. The development of resistance to chemotherapy in OC patients further complicates OC treatment outcomes^3^. Most OC patients respond well to platinum/taxane combination therapy, nevertheless, 75% of those relapse within the course of 2 years^4^. These statistical data regarding OC morbidity and mortality has been almost constant for many decades, which necessitates developing more efficient therapeutic strategies for OC treatment.

Over the last decade, high initial response rates were achieved by the standard treatment strategy of cytoreductive surgery followed by a taxane-platinum combination therapy, however, the majority of patients subsequently developed resistance^5^. Taxane (Tx) family is one of the most useful and effective antineoplastic agents including paclitaxel (taxol; PTX). It is usually combined with carboplatin to treat many types of cancer^6^. PTX's success in cancer therapy is due to its broad spectrum of antitumor activity in addition to its unique mechanism of action. PTX is a microtubule-stabilizing agent that selectively binds to microtubules and disrupts their dynamics, inhibiting chromosome segregation and subsequently causes mitotic arrest that leads to cell death^7^. Due to its high efficiency, PTX now is an FDA-approved drug as the first line treatment for OC in combination with platinum-based drugs (eg. Cisplatin). Despite the initial high response rates of OC to platinum/taxane treatment, around 80% of OC patients develop resistance to PTX^8^. The cellular and molecular mechanisms that lead to taxol resistance have been the subject of study for many years, yet still remains largely unknown.

MicroRNAs (miRNAs) are short RNA molecules of about 22 nucleotides in length, modulate the expression of almost all genes mostly on the post-transcriptional level. Tumor-suppressor genes, as well as oncogenes, lie under miRNA control, which makes the dysregulation in miRNA expression a cornerstone in cancer progression^9^. A growing body of evidence demonstrate the involvement of miRNAs’ dysregulation in the development of OC^10,11^. For example, high expression of miRNA-1207 was correlated with enhancing cancer stem cell-like features in OC through activation of the Wnt/beta-catenin signaling pathway^12^. In fact, many reports attributed the dysregulation of miRNA expression and the subsequent cancer progression to alterations in epigenetic marks at miRNA genes. The epigenetic landscape is known to be dramatically altered in cancer cells, affecting the expression of many coding and non-coding genes, including miRNAs^13^. For example, DNA hypermethylation was found to cause downregulation in miRNA-let-7a with subsequent upregulation of its oncogenic target IGF-II which led to poor prognosis in OC patients^14^. Interestingly, cancer cells acquiring chemoresistance are also known to epigenetically deviate away from their original chemosensitive precursors. The epigenetic silencing of miRNA-199b-5p has been reported to activate JAG1-Notch1 signaling leading to acquiring cisplatin resistance in OC cells^15^. Notably, the interrelation of epigenetics, miRNAs and PTX resistance triad has not been investigated in OC.

In this study, we used serous ovarian carcinoma cell line, KF-28, to map out the role/s of the epigenetically downregulated miRNAs in PTX resistance in OC. We merged data from high-throughput miRNAs and cDNA microarray profiling in both PTX-sensitive and PTX-resistant OC cells coupled with Gene Ontology (GO) and Kyoto Encyclopedia of Genes and Genomes (KEGG) database analysis and data mining to identify candidate miRNAs that can induce PTX resistance in OC with hyperfocus on those which are possibly epigenetically controlled. We also overexpressed or inhibited the miRNAs yielded by the pathway analysis and validated their correlation with their predicted signaling pathways and overall impact on OC response to PTX.

## Results

### PTX resistant cells show differentially expressed miRNAs profile

**Figure 1:**
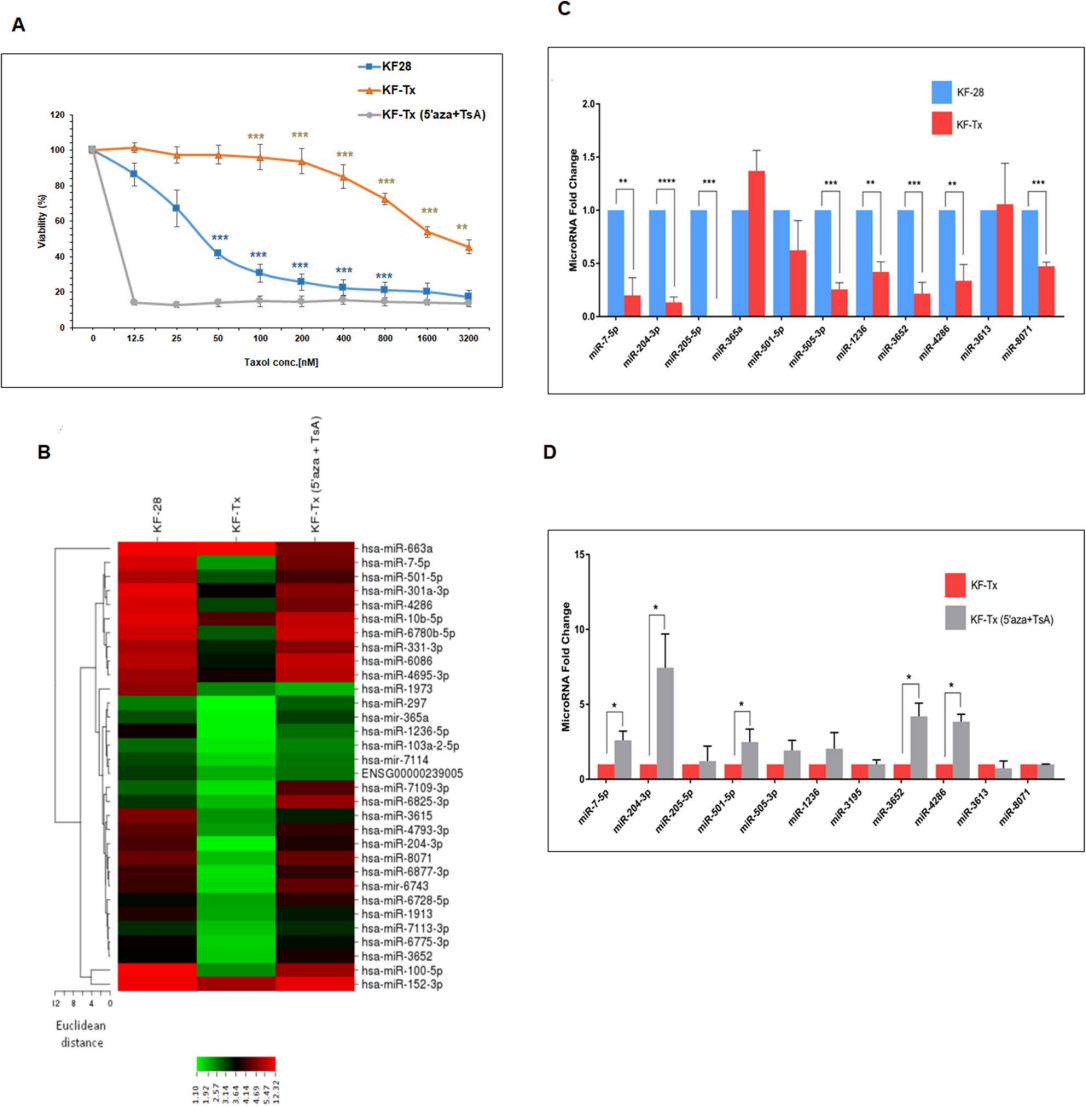
MiRNA profiling of taxol responsive (KF-28) and resistant (KF-Tx) OC cells. (**A**) Confirmation of chemoresistance of KF-Tx cells to PTX. The figure shows the differential response to PTX and the ability of the epigenetic modulators to restore sensitivity to PTX. (**B**) Heat map showing differentially expressed miRNAs in the PTX responsive, KF-28, cells versus PTX-resiatnt, KF-Tx, cells, and in KF-Tx cells versus KF-Tx cells following tratement with 5′AZA and TsA. (**C**) QPCR analysis result of the selected micorRNAs to confirm their downregulation in KF-Tx cells. (**D**) Relative expression of the same set of miRNAs in KF-Tx cells before and after co-treatment with 5′AZA (10 µM; 3 days) and TsA (300 nM; one day). U6 was used as a control. Values in A, C and D are means ± SE from tree independent experiments. One way anlysis of variance (ANOVA) was performed to get the satsitical significance of diffrence at each point (*p < 0.05, **p < 0.01 and ***p < 0.001).

MiRNAs microarray profiling of the OC cell line, KF-28, and their taxol resistant counterpart, KF-Tx (diffrencial response was validated by viability assay; Fig. 1A and supplementary figure S.1), identified multiple upregulated and downregulated miRNAs whose expression is changed due to the acquired state of PTX resistance (Fig. 1B). In this study, we are primarily interested in those miRNAs whose expression was possibly controlled by epigenetic mechanism/s. Therefore, we focused on the most downregulated miRNAs in KF-Tx cells when compared with the KF-28 cells. This comparison yielded a considerable number, 157, of miRNAs that were downregulated in KF-Tx cells. We subsequently narrowed down the list by only selecting miRNAs whose expression levels were re-elevated again in KF-Tx cells after treatment with the epigenetic modulators 5-Aza-2-deoxycytidine (5′AZA) and trichostatin A (TsA). Importantly the combinatorial treatment with 5′AZA/TsA with PTX significantly sensitized the resistant cells to PTX (Same Fig. 1A). Comparing expression profiles of miRNAs in KF-Tx cells before and after co-treatment with the epigentic modulators only revealed that the expression levels of miRNA-7-5p, miRNA-204-3p, miRNA-365a, miRNA-501-5p, miRNA-3652, miRNA-4286, and miRNA-8071 were restored with relative expression changes more than two folds (Fig. 1C and Table 1). These results suggest that these miRNAs might be candidates for hypermethylation and/or deacetylation at their promoter due to chemoresistance development.

**Table 1:** Microarray data of differentially expressed miRNAs.

MiRNA ID	Accession	Fold change (KF-28 vs. KF-Tx)	Fold change (KF-Tx vs. KF-Tx*)
hsa-miRNA-7-5p	MIMAT0000252	− 13.44	+ 4.5
hsa-miRNA-181c-3p	MIMAT0004559	− 13.01	
hsa-miRNA-181d-5p	MIMAT0002821	< − 20	
hsa-miRNA-3652	MIMAT0018072	− 3.73	+ 4.08
hsa-miRNA-204-3p	MIMAT0022693	− 7.58	+ 5.62
hsa-miRNA-155-5p	MIMAT0000646	< − 20	
hsa-miRNA-29c-3p	MIMAT0000681	− 19.45	
hsa-miRNA-455-5p	MIMAT0003150	− 16.27	
hsa-miRNA-29b-3p	MIMAT0000100	< − 20	
hsa-miRNA-200c-3p	MIMAT0000617	< − 20	
hsa-miRNA-4286	MIMAT0016916	− 7.09	+ 2.53
hsa-miRNA-31-5p	MIMAT0000089	< − 20	
hsa-miRNA-365a	MI0000767	− 3.39	+ 3.7
hsa-miRNA-8071	MIMAT0030998	− 5.9	+ 5.93
hsa-miRNA-31-3p	MIMAT0004504	< − 20	
hsa-miRNA-501-5p	MIMAT0002872	− 4.71	+ 2.17
hsa-miRNA-505-3p	MIMAT0002876	− 6.31	
hsa-miRNA-4448	MIMAT0018967	< − 20	
hsa-miRNA-3613-5p	MIMAT0017990	< − 20	
hsa-miRNA-1236-5p	MIMAT0022945	− 5.18	+ 2.94
hsa-miRNA-205-5p	MIMAT0000266	< − 20	
hsa-miRNA-6743	MI0022588	− 5.9	
hsa-miRNA-221-5p	MIMAT0004568	− 20	

KF-Tx* KF-Tx cells treated with the epigenetic modulators 5′AZA and TsA.

### Validation of microarray data using qPCR

**Figure 2:**
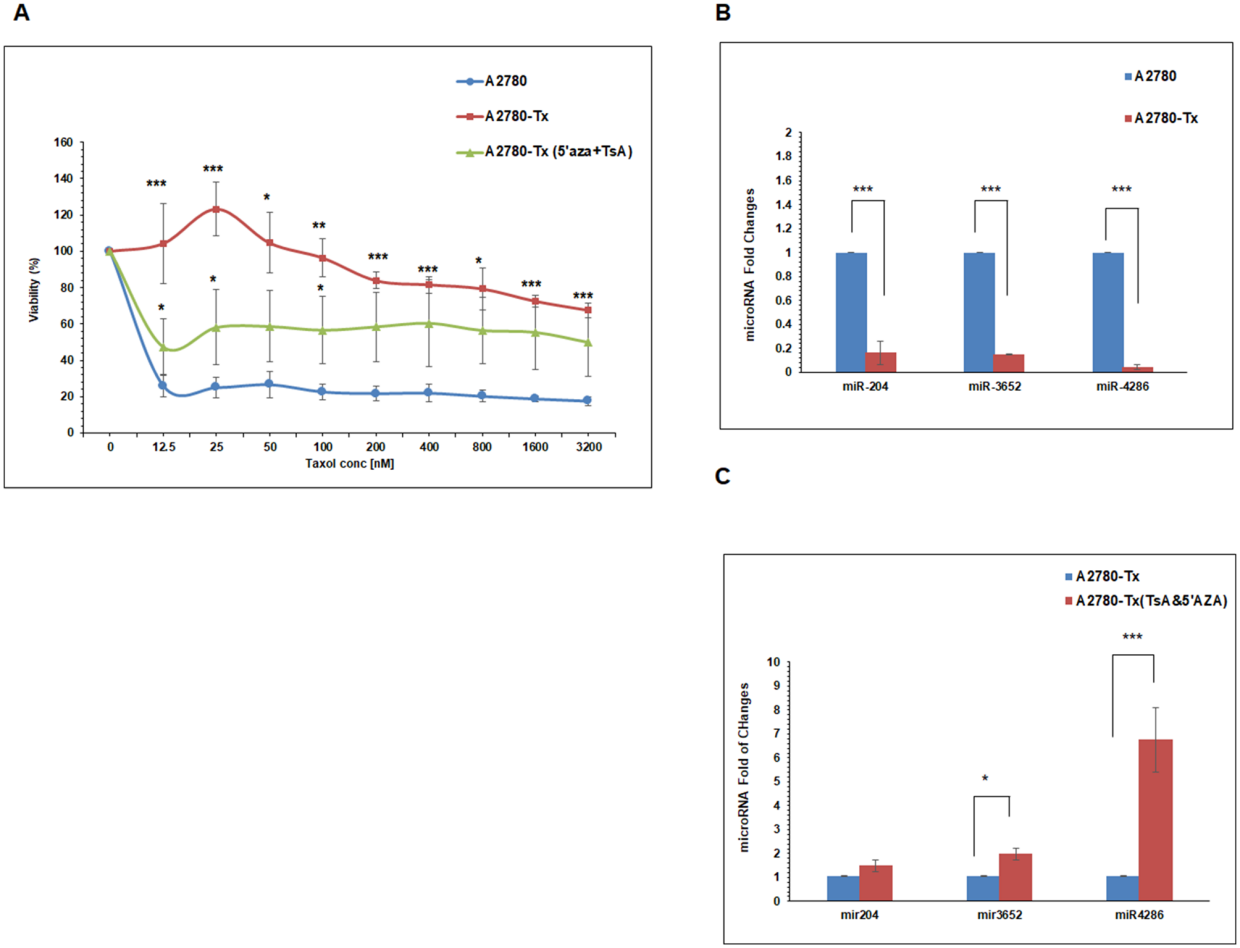
Differential expression of the selected miRNAs in A2780 and A2780-Tx cells. (**A**) Diffrential viability curve of the the taxol responsive A2780 cells versus taxol-resiatnt A2780-Tx cells and A2780 cells treated with epigenetic modulators (5′AZA and TsA) beside the indicated concentrations of PTX. (**B**) QPCR analysis of the selected micorRNAs in A2780 versus A2780-Tx cells. (**C**) Relative expression of the same set of microRs in A2780-Tx cells before and after co-treatment with 5′AZA (10 µM; three days) and TsA (300 nM; one day). U6 was used as a control. One way anlysis of variance (ANOVA) was performed to get the satsitical significance of diffrence at each point (*p < 0.05, **p < 0.01 and ***p < 0.001).

To validate microarray findings, we performed quantitative-PCR (qPCR) for those deregulated miRNAs listed above. Among the miRNAs that were downregulated in KF-Tx compared with KF-28 cells, miRNA-7-5P, miRNA-204-3P, miRNA-205-5P, miRNA-505-3P, miRNA-1236, miRNA-3652, miRNA-4286 and miRNA-8071 were confirmed by q-PCR (Fig. 1C) as significantly changed genes. In addition, we confirmed that miRNA-7-5P, miRNA-501-3P, miRNA-3652 and miRNA-4286 were significantly restored in KF-Tx cells after co-treatment with 5′AZA and TsA (Fig. 1D). To validate our observation in OC cells, we used another cellular set of OC A2780/A2780-Tx (Fig. 2A). Again, addition of the epigenetic modulators (5′AZA/TsA) to PTX, significantly enhanced response to PTX (Same Fig. 2A). The data indicated that the co-treatment significantly restores the sensitization of A2780-Tx cells to PTX treatment. Interestingly, the shortlisted miRNAs that showed the most reduction in expression then epigenetic-induced restoration in KF-Tx cells showed a similar expression pattern in A2780-Tx cells. MiRNA-204-3p, miRNA-3652 and miRNA-4286 were significantly downregulated in A2780-Tx cells compared with the parental responsive one, A2780 (Fig. 2B). However, these miRNAs were significantly re-upregulated in A2780 cells after co-treatment with 5′AZA and TsA, accordingly (Fig. 2C).

### Epigenetically regulated miRNAs modualte multiple signalling pathways in cancer

We next analysed the involvement of only the selected, epigenetically, regulated miRNAs in biological pathways with a specific focus on pathways involved in cancer development and progression. DIANA-miRNAPath tool was used with miRNA-7-5P, miRNA-501-3P, miRNA-3652 and miRNA-4286 as input. The validated miRNA targets from DIANA-TarBase v6.0 were pooled together and were linked to KEGG^16^ to identify the enrichment of these targets in all the biological pathways enlisted by KEGG. The analysis yielded a number of biological pathways that lie under the control of the given miRNAs and showed enrichment of miRNAs targets in multiple cancer-related pathways such as PI3K-Akt signaling pathway (70 genes), Wnt signaling pathway (27 genes) and ErbB signaling pathway (26 genes) (Table 2).

**Table 2:** KEGG pathways affected by epigenetically regulated miRNAs.

Pathway ID	Pathway description	*p*-value	Gene count	miRNA count
4520	Adherens junction	5.60E−07	27	3
4390	Hippo signaling pathway	1.76E−06	39	4
5200	Pathways in cancer	7.52E−05	90	4
4068	FoxO signaling pathway	0.000961137	39	4
4012	ErbB signaling pathway	0.010362241	26	3
4810	Regulation of actin cytoskeleton	0.019787554	46	4
4810	Wnt signaling pathway	0.03421743	26	4
4151	PI3K-Akt signaling pathway	0.042836907	70	4
4151	Proteoglycans in cancer	1.76E−06	49	4
4066	HIF-1 signaling pathway	0.005876187	30	3

### Epigenetically regulated miRNAs are involved in taxane resistance in OC

**Figure 3:**
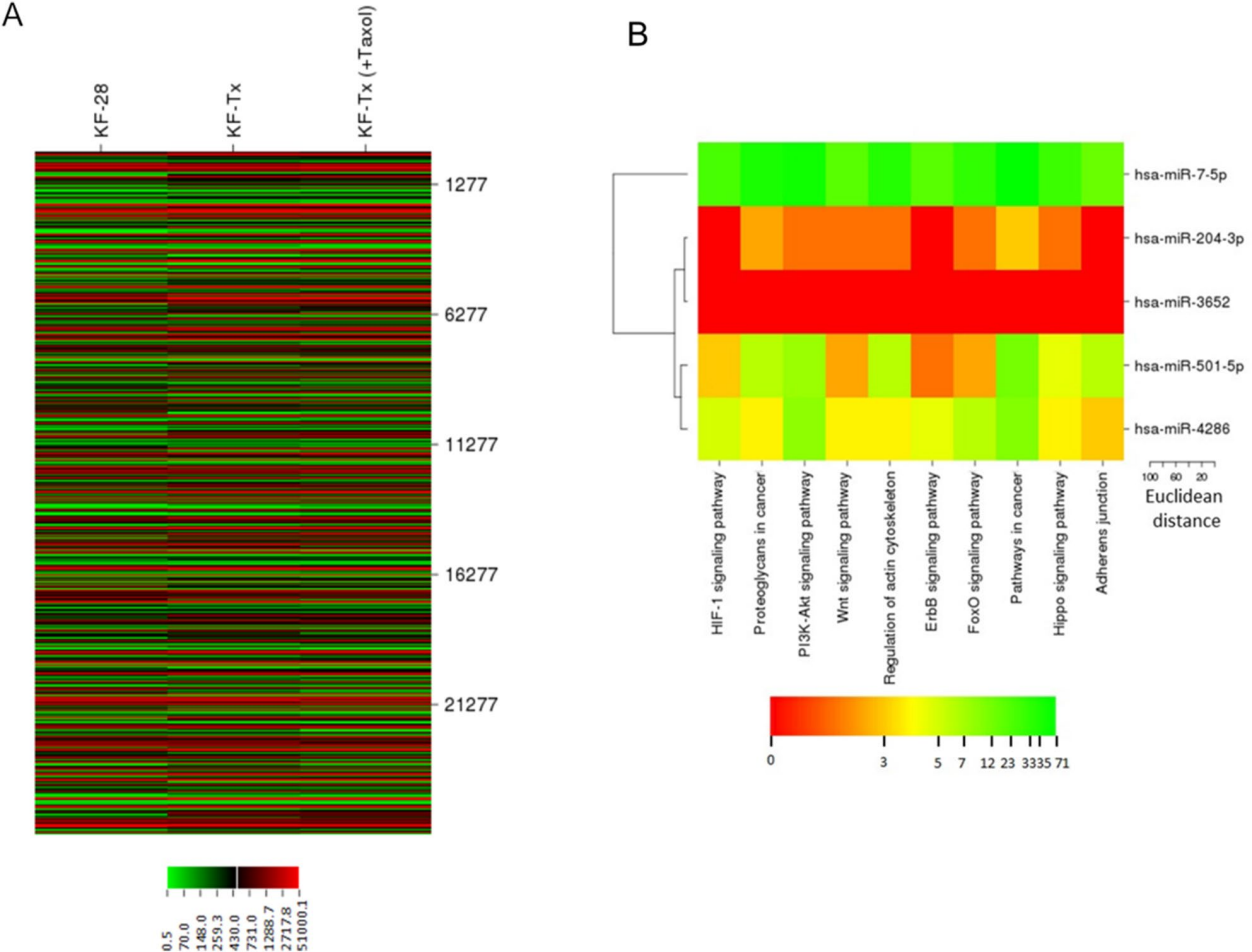
MRNA profiling of taxane responsive and taxane resistant OC cells. (**A**) Heatmap of a mRNA expression microarray analysis illustrating differential expression of genes in taxol-responsive cells (KF-28), taxol-resistant cells (KF-Tx) and KF-Tx cells treated with PTX. (**B**) Categorization of the miRNAs target genes and their pathways in KF-Tx before and after co-treatment with 5′AZA and TsA.

**Figure 4:**
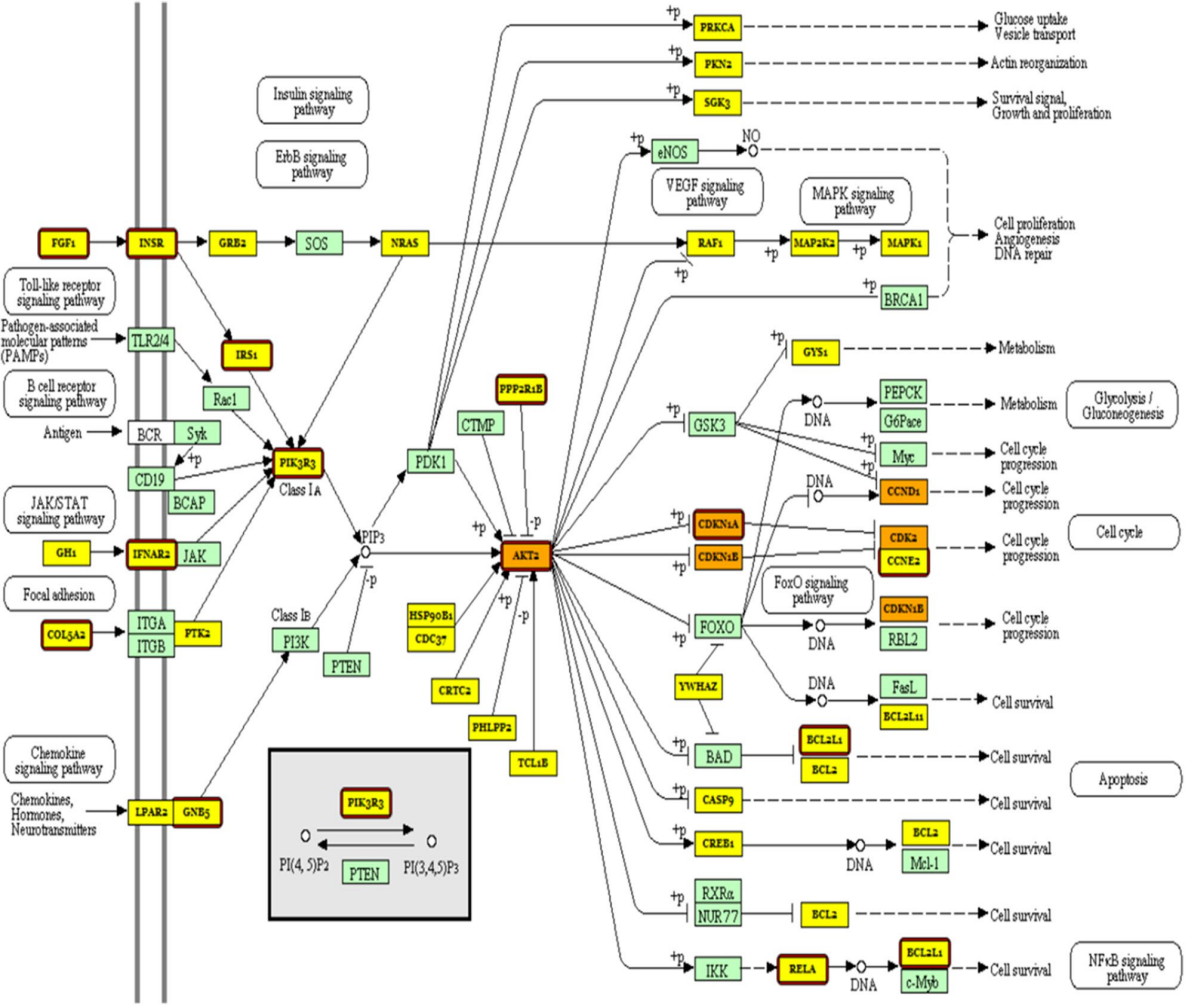
Pathway analysis of the selected miRNAs target genes in KF-Tx cells. Target genes of the selected miRNAs are clustered into pathways using KEGG database^16^ .Genes targeted by one miRNA (yellow boxes) and genes targeted by more than one miRNA (orange boxes) are shown. Multiple key genes that are upregulated in KF-Tx cells are shown in red-framed boxes. Green color boxes indicate genes which are not target for selected miRNAs.

In order to link the results of the aforementioned miRNAs’ targets, we set out to check the expression of the miRNA target genes indicated/validated in DIANA-miRNAPath in our cellular model of PTX resistance. We, therefore, performed a microarray analysis to compare the expression of over 25,000 genes in KF-28 and KF-Tx cells in addition to KF-Tx cells treated with a sublethal dose of PTX. The microarray analysis revealed hundreds of differentially expressed genes (Fig. 3A). We then merged the results from miRNA microarray with those from cDNA microarray, with a special focus on genes that are upregulated in KF-Tx compared to the KF-28 cell, as their upregulation could be attributed to the observed epigenetic downregulation of any of the identified miRNAs. This analysis revealed multiple oncogenes to be upregulated in KF-Tx cells compared to KF-28 (> 1.5 folds). The miRNAs target genes and their pathway in KF-Tx before and after co-treatment with 5′AZA and TsA were then categorized (Fig. 3B). This list includes key genes such as AKT2; V-akt murine thymoma viral oncogene homolog 2 (3.1 folds), CDKN1A; Cyclin D2 (5.68 folds), FGF2; Fibroblast growth factor 2 (5.2 folds) and PIK3R3; Phosphoinositide-3-kinase, regulatory subunit 3 (gamma) (1.5 folds) (Table 3). Applying gene lists into the KEGG database revealed roles in detrimental pathways that control cancer cell proliferation and survival such as PI3K-AKT pathway and MAPK-pathway in chemoresistance of OC (Tables 4, 5). Figure 4 illustrates an example where the PI3K-AKT signaling pathway detected by DIANA-miRNAPath to be targeted by miRNA-7-5P, miRNA-501-3P, miRNA-3652 and miRNA-4286, collectively. Genes targeted by one miRNA (yellow boxes) and genes targeted by more than one miRNA (orange boxes) are shown. Interestingly, multiple key genes in this pathway are upregulated in KF-Tx cells (Red-framed boxes) as indicated from cDNA microarray results. Many of those are well-known to control cell cycle and cancer development and progression. Of course, the miRNAs used in the pathway analysis do not affect the pathways listed evenly. The five miRNAs have different number of targets and thus affect a different signaling pathways with different intensities (Fig. 4) as illustrated by the heat map (Fig. 3).

**Table 3:** Upregulated genes in KF-Tx cells matched in DIANA-miRNAPath pathway analysis.

Gene ID	Gene name	Fold change	*p*-value
AKT2	V-akt murine thymoma viral oncogene homolog 2	3.1^a^	0.037598
FZD3	Frizzled family receptor 3	2.72	0.000732
CDKN1A	Cyclin-dependent kinase inhibitor 1A	5.68	0.000244
APC	Adenomatous polyposis coli	3.2^a^	0.000244
LAMC1	Laminin, gamma 1 (formerly LAMB2)	1.6	0.000244
CCND2	Cyclin D2	6	0.000244
GNB5	Guanine nucleotide binding protein (G protein)	1.84	0.000244
PIK3R3	Phosphoinositide-3-kinase, regulatory subunit 3 (gamma)	1.5	0.001953
PVRL1	Poliovirus receptor-related 1	1.68	0.030273
FGF2	Fibroblast growth factor 2	5.2	0.000732
WNT5A	Wingless-type MMTV integration site family, member 5A	3.2	0.000244
PAK1	P21 protein (Cdc42/Rac)-activated kinase 1	1.59	0.000732
CRK	V-crk sarcoma virus CT10 oncogene homolog (avian)	1.53^a^	0.000732
FADD	Fas (TNFRSF6)-associated via death domain	1.57	0.000244
BCL2L1	BCL2-like 1	1.58	0.000244
IRS1	Insulin receptor substrate 1	1.8	0.000244
PPP2R1B	Protein phosphatase 2, regulatory subunit A, beta	1.6^a^	0.000244
CFLAR	CASP8 and FADD-like apoptosis regulator	2.21	0.001953
FGFR3	Fibroblast growth factor receptor 3	1.9	0.000244
IFNAR2	Interferon (alpha, beta and omega) receptor 2	2.05	0.000732
CASP8	Caspase 8, apoptosis-related cysteine peptidase	1.83^a^	0.000732
PLCG2	Phospholipase C, gamma 2 (phosphatidylinositol-specific)	1.72	0.000244
CSNK1E	Casein kinase 1, epsilon	1.76	0.030273
TEAD1	TEA domain family member 1	1.6^a^	0.000244
E2F2	E2F transcription factor 2	3.34	0.010742
GPC4	Glypican 4	2.7	0.00293
RUNX2	Runt-related transcription factor 2	3.17^a^	0.023926
RELA	V-relreticuloendotheliosis viral oncogene homolog A	1.77	0.001221
VCL	Vinculin	1.78	0.000244

^a^Indicate that the indicated expression takes place only when KF-Tx cells were challenged with PTX. However, the other genes’ expression values (without asterisks) indicated changes in their expression in the resistant versus responsive cells without drug challenge.

**Table 4:** KEGG pathways and matched member genes.

Pathway/ID	Pathway description	Gene count	False discovery rate	Matching proteins in our network (labels)
5200	Pathways in cancer	15	4.07E−17	AKT2,BCL2L1,CASP8,CDKN1A,CRK,E2F2,FADD,FGF2,FGFR3, FZD3,LAMC1,PIK3R3,PLCG2,RELA,WNT5A
4151	PI3K-Akt signaling pathway	15	4.25E−17	AKT2,BCL2L1,CCND2,CDKN1A,FGF2,FGFR3,GNB5,IFNAR2,IRS1, LAMC1,PIK3R3,PPP2R1B,RELA
4014	Ras signaling pathway	9	1.77E−09	AKT2,BCL2L1,FGF2,FGFR3,GNB5,PAK1,PIK3R3,PLCG2,RELA
4210	Apoptosis	7	1.77E−09	AKT2,BCL2L1,CASP8,CFLAR,FADD,PIK3R3,RELA
4390	Hippo signaling pathway	7	4.85E−08	CCND2,FZD3,PPP2R1B,TEAD1,WNT5A
4012	ErbB signaling pathway	6	5.99E−08	AKT2,CDKN1A,CRK,PAK1,PIK3R3,PLCG2
4510	Focal adhesion	7	2.61E−07	AKT2,CCND2,CRK,LAMC1,PAK1,PIK3R3,VCL
5215	Prostate cancer	5	1.97E−06	AKT2,CDKN1A,E2F2,PIK3R3,RELA
4068	FoxO signaling pathway	5	9.19E−06	AKT2,CCND2,CDKN1A,IRS1,PIK3R3
4110	Cell cycle	5	9.27E−06	CCND2,CDKN1A,E2F2
4010	MAPK signaling pathway	6	1.50E−05	AKT2,CRK,FGF2,FGFR3,PAK1,RELA
4630	Jak-STAT signaling pathway	5	2.48E−05	AKT2,BCL2L1,CCND2,IFNAR2,PIK3R3
4910	Insulin signaling pathway	4	0.000261	AKT2,CRK,IRS1,PIK3R3
4310	Wnt signaling pathway	4	0.00028	CCND2,FZD3,GPC4,WNT5A
4150	mTOR signaling pathway	3	0.000505	AKT2,IRS1,PIK3R3
4115	p53 signaling pathway	3	0.000646	CASP8,CCND2,CDKN1A

**Table 5:** GO pathways and matched member genes.

Pathway ID	Pathway description	Gene count	False discovery rate	Matching proteins in your network (labels)
0007165	Signal transduction	24	1.72E−07	BCL2L1,CASP8,CDKN1A,CFLAR,CRK,CSNK1E,E2F2, FADD,FGF2,FZD3,GNB5,GPC4,IFNAR2,IRS1,PAK1, PIK3R3,PLCG2,PVRL1,RELA,RUNX2,TEAD1,WNT5A
0050776	Regulation of immune response	12	5.70E−07	CASP8,CDKN1A,CRK,FADD,FGF2,FGFR3,IFNAR2, IRS1,PAK1,PLCG2,RELA,WNT5A
0043549	Regulation of kinase activity	11	2.04E−06	APC,CCND2,CDKN1A,CRK,FGF2,FGFR3,IRS1,PAK1, PIK3R3,WNT5A
0097190	Apoptotic signaling pathway	8	8.93E−06	BCL2L1,CASP8,CFLAR,E2F2,FADD,FGFR3
0010941	Regulation of cell death	13	1.10E−05	AKT2,BCL2L1,CDKN1A,CFLAR,FGF2,FGFR3,FZD3, PAK1,PLCG2,PPP2R1B,RELA
0008286	Insulin receptor signaling pathway	6	2.41E−05	AKT2,CRK,FGF2,FGFR3,IRS1,PIK3R3
0006915	Apoptotic process	11	2.89E−05	AKT2,APC,CASP8,CFLAR,E2F2,FADD,FGFR3,PAK1, PPP2R1B
0043066	Negative regulation of apoptotic process	10	3.29E−05	AKT2,APC,BCL2L1,CASP8,CDKN1A,CFLAR,FADD, FZD3,RELA,WNT5A
0043551	Regulation of phosphatidylinositol 3-kinase activity	4	4.91E−05	FGF2,FGFR3,IRS1,PIK3R3
0008284	Positive regulation of cell proliferation	9	0.000156	BCL2L1,CDKN1A,FADD,FGF2,FZD3,IRS1,LAMC1, RELA,RUNX2
0030335	Positive regulation of cell migration	6	0.000777	AKT2,APC,FADD,FGF2,IRS1,WNT5A

### Protein–protein interaction network analysis

**Figure 5:**
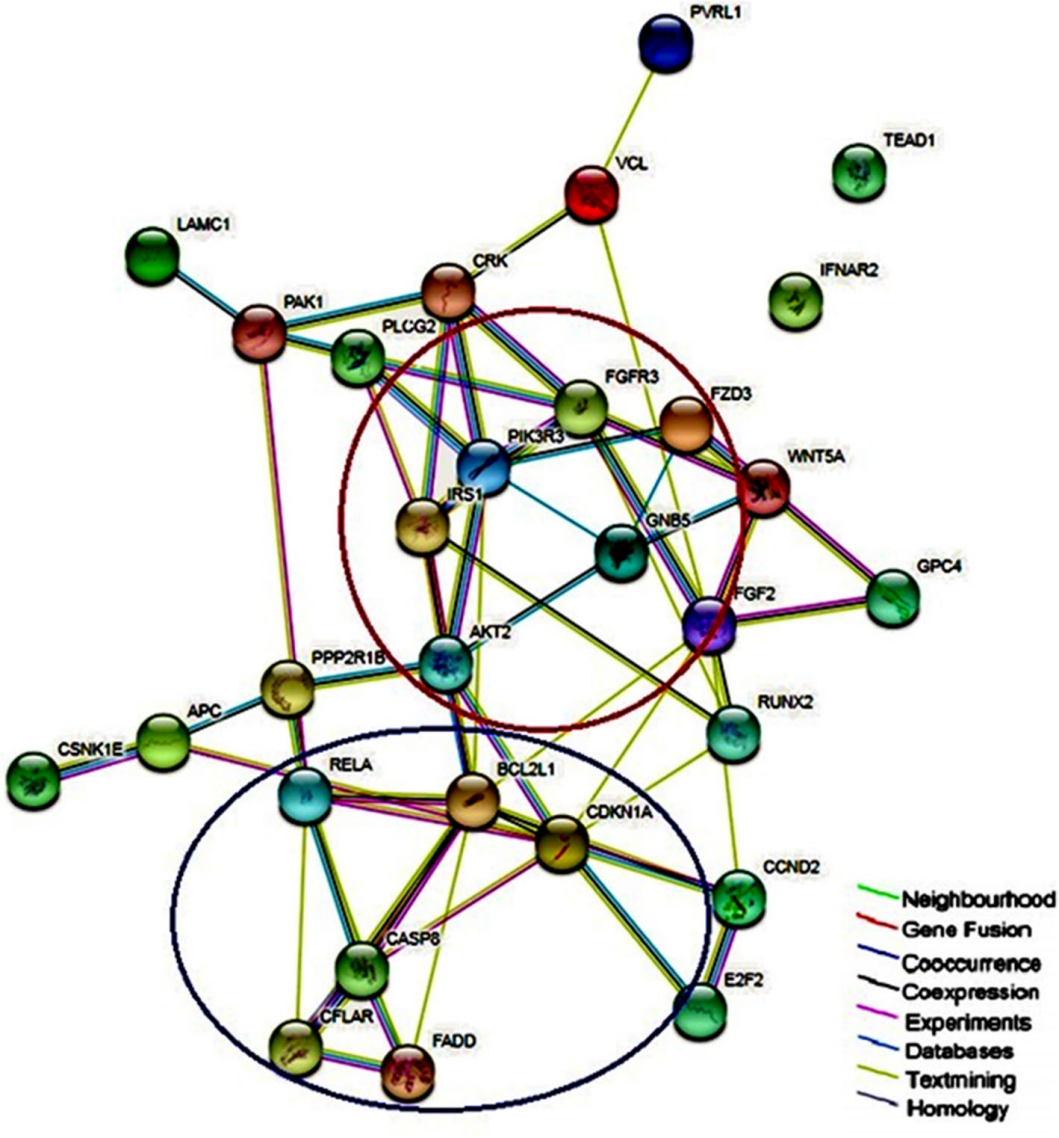
Protein–protein interaction (PPI) network of differentially expressed miRNAs’ target genes. The shortlisted miRNAs targets were subjected to Search Tool for the Retrieval of Interacting Genes (STRING) 10.5 database analyses. STRING constructed a network model showing all protein interactions, which islinked to KEGG and Gene Ontology (GO) databases to cluster the shortlisted genes as the input into pathways and biological processes.

We next analyzed the upregulated genes in KF-Tx cells that were matched in KEGG and GO databases using the STRING software. The predicted protein–protein associations were queried through a vast number of databases derived in different ways, such as experimentally determined interactions, protein neighborhood data, or data acquired via text mining. For the predicted 29 upregulated proteins, two main networks of protein interactions were identified, including Growth Factor-induced PI3K-Akt signaling network (red circle) and anti-apoptotic network (Blue circle) (Fig. 5).

### Epigenetically regulated miRNAs sensitize OC to PTX

**Figure 6:**
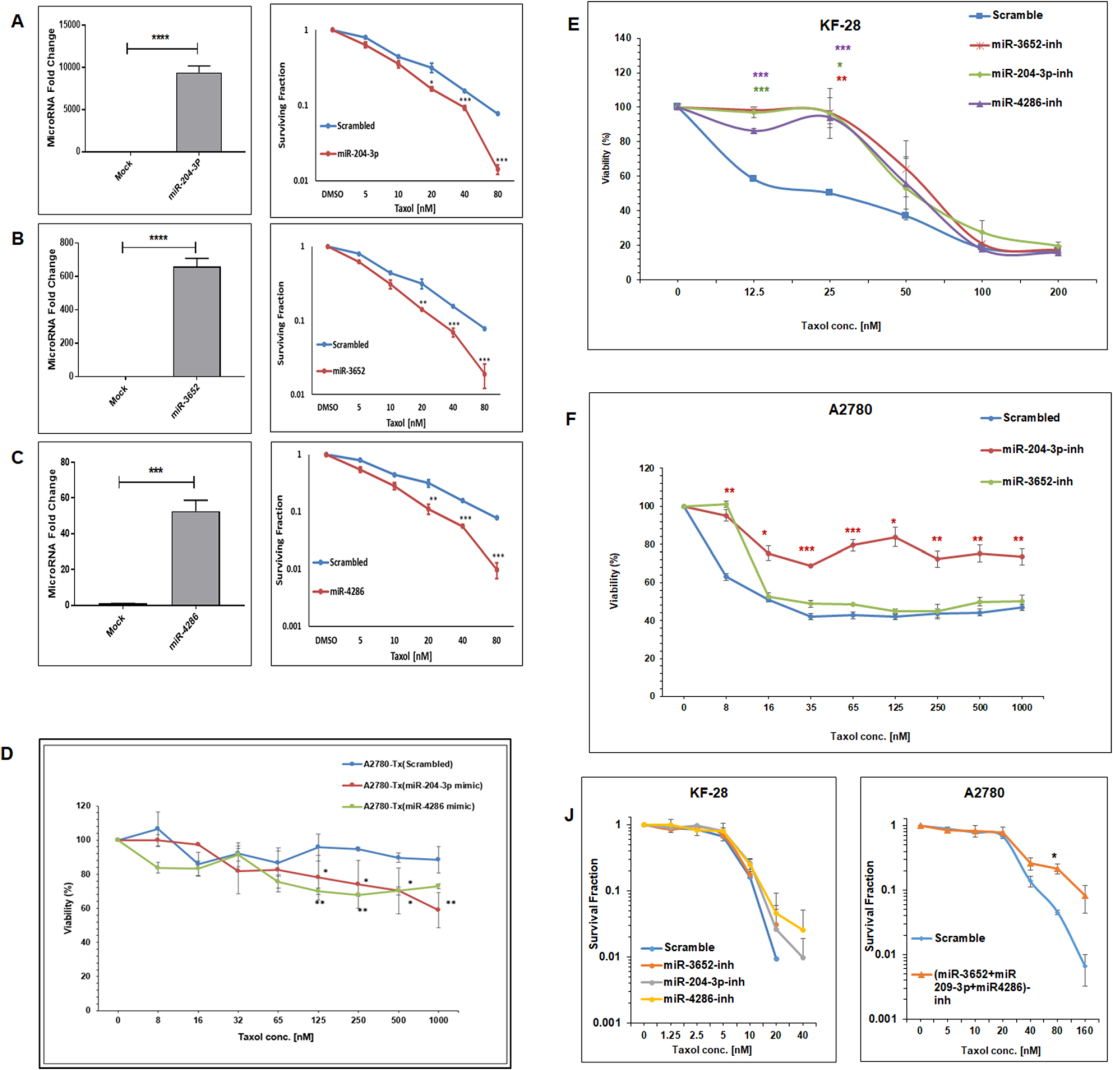
Overexpression of the epigenetically regulated miRNAs restors OC cells’ response to PTX. Quantitative PCR and **c**olony formation assay for KF-Tx cells transfected with (**A**) micoRNA-204-3p, (**B**) miRNA-3652, and (**C**) MiRNA-4286 mimics, respectively. The left panels are qPCR of each gene validating the over-expression while the left panels are colony forming ability results from each miRNA transfected or scrambeled. (**D**) Viability assay results from A2780-Tx cells after transfection of miRNA-204-3p, miRNA-4286 mimics or scambled RNA, treated with the indicated concentrations of PTX. Viability of the KF-28 (**E**) and A2780 (**F**), treated with the indicated concentrations of PTX, after transfection with indicated miRNA inhibitor/s or scramble. (**J**) Clonogenic assay results of KF-28 cells transfected with the indicated miRNA inhibitor/s or scramble RNA followed by treatment with the indicated concentrations of PTX (left panel) while similar results from A2789 cells (right panel), as the cells were treated with PTX after transfection with the mixture of the indicated miRNAs inhibitors followed by PTX. Results are means ± SE from three independent experiments. One way analysis of variance (ANOVA) was performed to get the statistical significance of difference at each point (*p < 0.05, **p < 0.01 and ***p < 0.001).

In order to confirm that the expression of the shortlisted miRNAs is functionally correlated with resistance of OC cells to PTX, colony formation assay was performed. The mimic-based overexpression of the different miRNAs was confirmed by qPCR (Fig. 6A–C; left panels). The overexpression of miRNA-204-3p, miRNA-3652 or miRNA-4286 resulted in significant sensitization of KF-Tx cells to PTX as identified by a reduction in the surviving fractions of the miRNA-transfected cells compared to controls (Fig. 6A–C, respectively, right panels). In addition, the mimic-based overexpression of two representative miRNAs (miRNA-204-3p and miRNA-4286) in A2780-Tx cells has significantly restored the sensitization to PTX as proved by viability assay (Fig. 6D). Additionally, we validate our observation by inhibiting the selected three miRNAs (miRNA-3652, miRNA-204-3p. and miRNA-4286) in the parental, KF-28, cells. Transfection of the KF-28 cells with miRNA/s inhibitor conferred resistance to KF-28 cells against PTX and enhanced their viability, specifically at concentration of 25 nm (Fig. 6E). Similar results were obtained from A2780 cells, especially those transfected with inhibitor for miRNA-204-3p (Fig. 6F). Again, these results were confirmed by colony forming assay when KF-28 cells were transfected with each miRNA inhibitor individually (Fig. 6J, left panel), and when A2780 cells were transfected with mixture of the three miRNA inhibitors (Fig. 6J, right panel). The results revealed that the indicated miRNAs inhibition significantly induce PTX resistance in both responsive KF-28 and A2780 cells. Also, These results verify the involvement of those epigenetically controlled miRNAs in the PTX resistance in OC cells.

**Figure 7:**
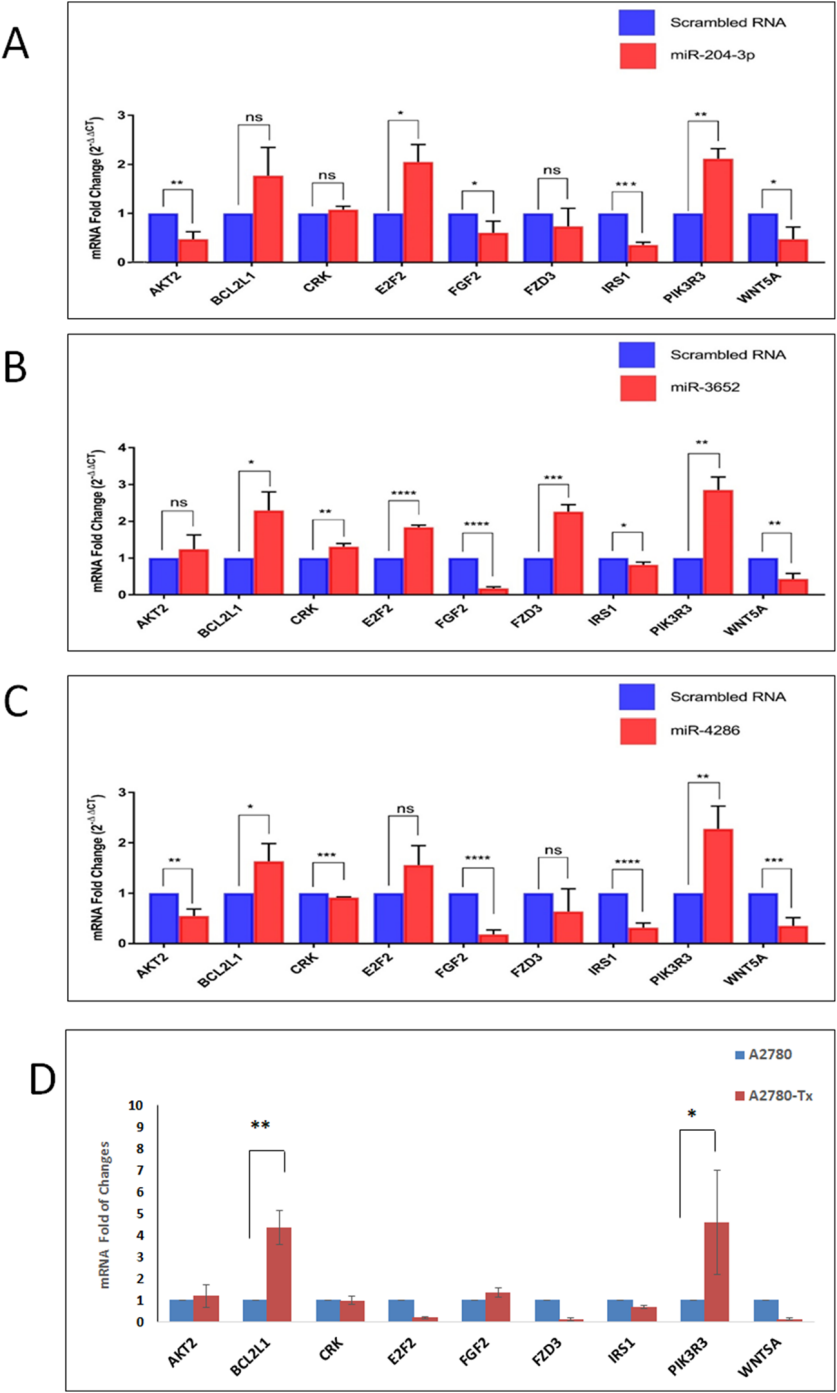
Overexpression of the epigenetically regulated miRNAs affects the expression of predicted downstream targets. qPCR showing variation in expression of miRNAs’ target genes in response to the indicated miRNAs overexpression of (**A**) miRNA-204-3p, (**B**) miRNA-3652, and (**C**) miRNA-4286 in KF-Tx. (**D**) qPCR showing diffrencial expression of the same miRNAs’ target genes in A2780 cells and A2780-Tx cells. The results are means ± SE from independent triplicates (*p < 0.05, **p < 0.01 and ***p < 0.001) as obtained from ANOVA.

Moreover, we chose one representative miRNA-4286, and confirmed that its overexpression in the KF-Tx cells significantly enhanced the annexin V positive population accumulation, as an indication for the early apoptotic cells, when cells were treated with PTX for 36 h at 500 nM (Supplementary figure S.2). To further determine the potential effect of each of those selected miRNAs on the target genes, qPCR analysis was performed after overexpression of the miRNAs in KF-Tx cells. Overexpression of miRNA-204-3p led to significant downregulation of AKT2, Fibroblast Growth Factor 2 (FGF2), IRS1 as well as Wnt5A mRNAs. In addition, overexpression of miRNA-3652 resulted in significant downregulation of FGF2, IRS2 and Wnt5a mRNAs. While overexpression of miRNA-4286 resulted in significant downregulation of AKT2, CRK, FGF2, IRS1 and Wnt5a mRNAs (Fig. 7A–C, respectively). Similar to the cDNA data from KF-28/KF-Tx set, some of these genes (BCL2L1 and PIK3R3) were differentially expressed in the A2780/A2780-Tx set of cells (Fig. 7D).

### AKT2 expression mediate the PTX response through indicated miRNAs

**Figure 8:**
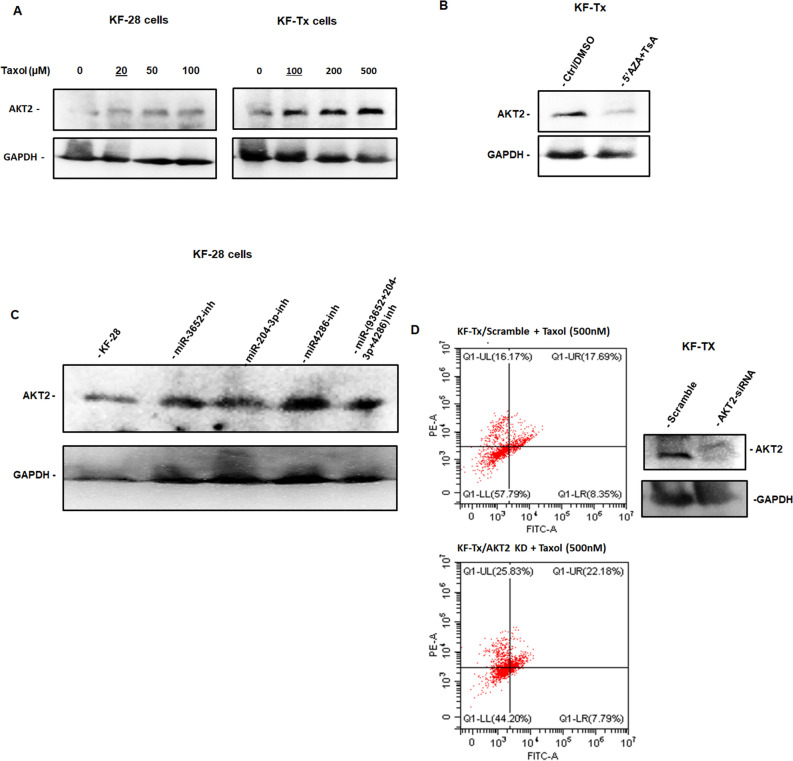
AKT2 expression mediates the response of KF-28 and KF-Tx and responses to epigentic modulators. (**A**) Western bloting result shows AKT2 expression in KF-28 and KF-Tx cells in response to PTX at the indicated concentrations. The underlined conc. Indicate the IC50 for each cell line. (**B**) Western bloting result indicating the downregulation of AKT2 in response to the combination of 5′AZA and TsA in KF-Tx cells. (**C**) Western bloting result indicating the expression of AKT2 in the KF-28 parental cells transfected with each single miRNA inhibitor, mixture of the three miRNA inhibitors or scrambled RNA together. (**D**) right panel indicates the downregulation of AKT2 in response to specific siRNA compared with control siRNA in KF-Tx cells. The left panel is annexin/PI results show the relative sensitization of KF-Tx cells to PTX after AKT2 knock down.

Previous reports showed evidence that supports our qPCR data and indicates that the upregulation on AKT2 expression induces OC cells to resist PTX^17^. To further support our findings, we investigated the expression of AKT2 protein by western blotting in both KF-28 and KF-Tx cells treated with different concentrations of PTX (starting from concentration equivalent to IC50 of each cell line) for 36 h. In accordance with cDNA microarray data, our protein analysis shows differential basic expression of AKT2 at zero (0;vehicle) concentration of PTX. In addition, AKT2 expression was enhanced in response to PTX in both KF-28 and KF-Tx cells in a dose-dependent manner (Fig. 8A). Whereas the co-treatment by the epigenetic modulators; 5′AZA and TsA suppressed AKT2 expression in KF-Tx cells (Fig. 8B). Interestingly, we found that the inhibition of the shortlisted miRNAs individually or in combination restored AKT2 expression in KF-28 cells (Fig. 8C). Moreover, siRNA-based silincing of AKT2 enhanced the response to PTX as indicated by annexin/PI staining (Fig. 8D).

## Discussion

Expression profiling has been widely used to reveal the abnormal gene regulation associated with OC^18^ and has enabled the identification of potential targets for improved therapeutic strategies. Understanding the mechanism behind such expression profile changes would help to improve treatment strategies to decrease the incidence of chemoresistance and thus improve patients’ prognosis. Here, we hypothesized that the upregulation of some genes involved in chemoresistance is due to epigenetic-mediated downregulation of key cellular miRNAs. The analysis of miRNA expression profile as well as mRNA profile of a representative chemoresponsive versus chemoresistant OC cell line before and after treatment with global epigenetic modulators not only helped us to map out the major mechanisms involved in chemoresistance but also shed light to those miRNAs which might be epigenetically controlled during resistance development. The putative target mechanism/s was then analyzed in silico, which provided new insights into OC resistance.

It is well established that cancer epigenome significantly contributes to the pathogenesis of many cancers including OC^19^. DNA methylation and/or histone deacetylation are regulators of miRNAs expression and may contribute to the development of chemoresistance. The silencing through DNA methylation can be reversed by demethylating drugs like 5′AZA while silencing through histone deacetylation can be reversed by global HDAC inhibitors like TsA. Both drugs act in synergy to reverse the transcriptionally/epigenetically repressed genes, a strategy previously described in many tumor types^20^.

The fact that combinatorial treatment of 5′AZA and a low dose of PTX was well tolerated and led to favorable clinical outcomes in chemoresistant OC patients^21^ supports our experimental approach of targeting the drug-induced variations in epigenomic landscape in PTX-resistant OC cells. At first, we assessed the differential expression of miRNAs in the OC cell line, KF-28, and its PTX resistant counterpart, KF-Tx, before and after co-treatment with 5′AZA and TsA. We focused on miRNAs that were downregulated in KF-Tx cells when compared with the parental one proposing that this downregulation might be due to epigenetic reasons. Microarray analysis revealed 157 miRNAs to be downregulated in KF-Tx cells. Among the top 25 downregulated miRNAs, 7 miRNAs were restored to significantly higher expression levels when this resistant, KF-Tx, cells were co-treated with 5′AZA and TsA. Results from qPCR analysis confirmed the array data and narrowed down the list to five miRNAs; miRNA-7-5p, miRNA-204-3p, miRNA-501-5p, miRNA-3652 and miRNA-4286. The fact that these five miRNAs were downregulated in PTX resistant cells suggests that they contribute to the sensitization of OC cells to PTX. In addition, the restoration of their expression after drug-induced epigenetic modification suggests that they are under tight epigenetic control in KF-Tx cells. These data are consistent with the large scale differential expression of miRNAs in PTX-resistant OC cells^22^. Notably, our study is the first to report a large-scale expression profile of epigenetically regulated miRNAs in a resistant OC model, which subsequently identifies five novel miRNAs as new players in OC resistance.

Our next aim was to analyze the possible role(s) of the five epigenetically regulated miRNAs in our chemoresistance model of OC. To achieve this, a complex miRNA pathway analysis was performed. We made use of the data-mining resource DIANA-miRNAPath software. DIANA-miRNAPath is a miRNA pathway analysis webserver that provides accurate statistics and can accommodate advanced search pipelines. miRNAPath can utilize experimentally validated miRNA interactions derived from DIANA-TarBase v6.0 and subsequently combine this data with sophisticated merging and meta-analysis algorithms^23^. A collective pathway analysis of the five shortlisted miRNAs yielded a list of over validated 2000 genes by DIANA-TarBase v6.0. This large number of genes is mainly accounted for by miRNA-7-5p which alone yielded 1,830 validated genes as it is one of the most heavily studied miRNA. The validation techniques include microarray, qPCR, western blot, PAR-CLIP and HITS-CLIP^23^. Next, we used the tool available by KEGG database as well as Gene Ontology (GO) analysis to cluster the list of validated miRNAs and their target genes into cellular pathways and biological processes (Table 2). The analysis shows that the PI3K/AKT pathway is the most affected signaling pathway by the five miRNAs where 70 genes, mediating this pathway, were significantly affected.

The cDNA microarray data shows the expression profile of over 25,000 genes in KF-Tx and KF-28 cells, to check the status of validated miRNA targets in this model. Interestingly, we found key genes in PI3K/AKT signaling pathway to be upregulated in KF-Tx cells (Table 3). The upregulated genes included PIK3Rs, AKT2, CDKN1A and CCND1, among others. It is thus reasonable to believe that the proposed miRNAs targeting these genes which are downregulated in PTX resistant cells allowing these target genes to be upregulated and to proceed with their oncogenic effects. Thus, our analysis proposes an indirect epigenetic regulation of this pathway through key upstream miRNAs. Furthermore, analysis of protein–protein association clustered the 29 upregulated genes into two major networks; PI3K/AKT signaling and an anti-apoptotic network that includes BCL2L1, CASP8, CDKN1A and RELA.

PI3K/AKT pathway is activated in approximately 70% OC^24^. Its activation is associated with higher invasive and migratory capacities^24^, making this pathway a potential predictor of invasiveness for ovarian tumor cells^25^. A prominent key member in this pathway, PIK3R3, phosphorylates and activates AKT2 which leads to propagation of the pathway^26^. Our findings go in line with previous studies which showed that silencing PIK3Rs hinders OC SKOV3 cells proliferation, migration, and invasion^27^. The protein–protein association analysis sets AKT2 at the center of the network. AKT2 is well known to induce anti-apoptotic processes. For example, AKT2 blocks the apoptotic function of BAD via phosphorylation^28^. Supporting to our data, a previous study revealed that overexpression of AKT2 stimulates the OC cells to override the PTX treatment^17^ while targeting AKT2 by siRNA sensitized OC cells to PTX^29^. It was also previously reported that AKT2 inactivation by siRNAs lead to anti-proliferative effect in OC cells OVCAR-3 and SKOV-3^30^. It was of great importance to show that all these in silico predicted alterations in key pathways would functionally reflect on the resistance phenotype of the PTX resistant cells. The ability of assessed miRNAs to reverse the resistance phenotype of the KF-Tx and A2780-Tx cells (Fig. 5A–D) through modulating AKT2 expression (Fig. 8A–D) proves the efficacy of the in silico tools used to track the targeted downstream pathways of those miRNAs.

In conclusion, this study sheds light on the role of epigenetic manipulation to reverse PTX resistance in OC. We made use of high throughput miRNA and cDNA array profiling coupled with pathway analysis and data mining to show that epigenetically regulated miRNAs can modulate major signaling pathways in PTX resistant OC cells. This work paves the way to more in-depth mechanistic studies, which is running in our laboratory now, for better understanding of each miRNA’s specific target, better illustration of the molecular players in PTX resistance, and to identify potential targets for therapy. Such future experiment will also assess whether epigenetic silencing of these genes is essential or only individual silencing is enough to develop resistance phenotype to representative from taxane family, PTX.

## Methods

### Antibodies (Abs) and reagents

Mouse anti-human AKT2 and mouse anti-human GAPDH were purchased from Santa Cruz (USA) Immunoblotting detection was done with anti-mouse secondary horseradish-peroxidase-conjugated antibodies (Dako). Abs were used at the indicated dilution (Western blotting section. Paclitaxel was supplied by ChemCruz (USA). The working stock was diluted in the media at a final concentration of 4 μM and further dilutions were carried out to reach the desired concentration for experiments.

### Cell culture and treatment

The human ovarian serous cancer cell line, KF-28, was kindly provided by Prof. Yoshihiro Kikuchi (Department of Obstetrics and Gynecology, National Defense Medical College, Saitama, Japan) who established this cell line from tissue of a patient with low grade serous cystadenscarcinoma of the ovary on November 20, 1982. This KF-28 cell line represented a very attractive model to conduct our study because it is a low-grade cell line with a wild-type TP53 gene which makes it a perfect choice for chemoresistance studies. That is because it is known that, low-grade parental cell line with a relatively low baseline IC50 value enables us develop a chemoresistant daughter cell line with a two to five folds increase in resistance with an IC50 value that still lies within the clinically relevant treatment range. It is worth noting that this cell line has wild-type TP53^31–34^. A2780 cells line was gifted by Prof. Hidemich Watari, Graduate School of Medicine, Hokkaido University, Japan as purchased from ECACC (ECACC 93112519). Both Cell lines were maintained in RPMI-1640 (Sigma-Aldrich, USA) supplemented with 2mML-glutamine (Lonza, Belgium) and 10% FBS (Sigma, St. Louis, MO, USA), 1% Penicillin/Streptomycin (Lonza, Belgium) at 37 °C in 5% CO2 atmosphere (NuAire). KF-Tx and A2780-Tx cells were established from parental cell lines, KF-28 and A2780, respectively, by maintaining them in increasing sublethal concentrations of PTX (up to10 nM) for more than 10 months. Detection of IC_50_ for each clone by 3-days viability assay was determined and demonstrated that IC_50_ of the KF-Tx cells showed almost eight-fold increase compared with the parental cells. For 5′AZA and TsA treatment, cells were grown in 10 cm^2^ culture dishes to a monolayer confluence of 70%. Cells were treated everyday with fresh media containing 10 µM 5′ AZA for three days while drug-containing media was replaced every day with the addition of 300 nM TsA in the third day followed by cell lysis or RNA purification.

### RNA extraction and microarray processing

For microarray and qPCR experiments, cells were grown in 10 cm^2^ culture dishes to a monolayer with confluence of 90% in triplicates. After different treatments, cells were washed twice with PBS and then pelleted by centrifugation for 5 min at 1800 rpm at 4 °C. Cells were lysed by Trizol reagent (Lonza, Belgium) and then total RNA was purified with phenol/chloroform extraction method. Concentration and purity of RNA samples were measured using Nanodrop (Qiagen; Germany). The RNA purity was assessed OD260/OD230 (≥ 1.5) using NanoDrop. gDNA contamination was evaluated by gel electrophoresis. The RNA from three biological samples was then pooled for miRNA analysis. The 2.5 μg and 13 μg of total RNA were individually determined for miRNA profiles and mRNA profiles using Human miRNAOneArray v4 chip andGenechip array, respectively(Affymetrix; USA). The chip uses two groups of probes respectively labeling with Cy3 and Cy5 fluorescent dyes to generate 532 nm and 635 nm excitation. The intensities of fluorescent were analyzed (Molecular Devices, Sunnyvale, CA). The miRNAs raw intensity of each probe was passed the criteria normalized by 75% median scaling normalization method. Target scan and miRNADB online database was further used in the annotation analysis for miRNAs target prediction and functional annotations.

### Quantification of expression using qPCR

To quantify the expression of miRNAs in the different cell types, MystiCq® miRNA cDNA Synthesis kit (Sigma-Aldrich, USA) was used to prepare cDNA for miRNAs in each RNA sample. This two-step process involves first polyadenylation on miRNAs by adding 2 ul Poly (A) Tailing Buffer (5X), 1 ul Poly (A) Polymerase to up to 7 ul of RNA and incubation at 37 °C for 60 min and 70 °C for 5 min. Then, a 10 ul cocktail containing 9 ul MystiCq miRNA cDNA Reaction Mix and 1 ul Reverse Transcriptase is added to the 10 ul polyadenylation reaction and incubated at 42 °C for 20 min, then at 85 °C for 5 min. QPCR quantification of miRNAs was done using the miScript SYBR Green PCR Kit (Qiagene, Germeny) according to manufacturer’s instructions. The primers used in the qPCR reaction involved a Universal Reverse primer supplied in the MystiCq® miRNA cDNA Synthesis kit (Sigma-Aldrich, USA) and a forward primer specific for each different miRNA assayed in addition to a forward primer specific for the house-keeping control RNU6B. For mRNA quantification, iScript cDNA Synthesis Kit(Bio-Rad, USA) was used to prepare cDNA from RNA samples according to manufacturer’s instructions. In brief, 4 ul 5× iScript Reaction Mix were mixed with 1 ul iScript Reverse Transcriptase, a volume of RNA sample equivelant to 200 ng RNAand the reactions were completed with Nuclease-free water to 20 ul. Reactions were then incubated in T100 Thermal Cycler (Bio-Rad, USA) as follow; 5 min at 25 °C, 20 min at 46 °C and finally 1 min at 95 °C. For qPCR, 10 µl Taq Universal SYBR Green Supermix (Bio-Rad, USA) were mixed with 2 µl cDNA, specific forward and reverse primers and the reaction volume was adjusted to 20 µl using Nuclease-free water. qPCR was reactions were run in the QuantStudio™ 12 K Flex system (Applied Biosystems, USA) and cycling conditions were as follows; 5 min at 95 °C followed by 40 cycles of 15 s at 95 °C and 1 min at 60 °C. CT values were then collected. mRNA expression levels were calculated using ΔΔCT method using GAPDH as housekeeping gene for normalization. Statistical significance of expression variations was calculated on Graphpad Prism using two-tailed Student T-test.

### MiRNA pathway analysis

A pathway analysis was performed for shortlisted miRNAs to predict the molecular mechanisms by which those miRNAs can alter the OC cells response to PTX. The analysis was done using the DIANA LAB tool miRNAPath. DIANA-miRNAPath incorporates an updated database of experimentally validated miRNA targets (DIANA-TarBase v6.0) which is able to combine all validated targets of the selected input miRNAs and perform a hierarchical clustering of miRNAs and their affected pathways based on their interaction levels. DIANA-miRNAPath is also linked to the Kyoto Encyclopedia of Genes and Genomes (KEGG) which classifies biological pathways^16,23^.

### Analysis of miRNAs target proteins interactions

In order to evaluate the protein–protein interactions of shortlisted miRNAs’ targets, Search Tool for the Retrieval of Interacting Genes (STRING) 10.5 database was used. The database includes both known and predicted direct physical or functional interactions between proteins. STRING constructed a network model showing all protein interactions in our protein set. STRING is also linked to KEGG and a Gene Ontology (GO) databases to cluster the shortlisted genes used as the input into pathways and biological processes.

### Colony formation and proliferation assay

The ability of the identified miRNAs to reverse the state of resistance of KF-Tx to PTX was assessed using colony formation assay. MiRNA mimics (Qiagen, Germany) for miRNA-204-3p, miRNA-3652 and miRNA-4286 were transfected into KF-Tx cells using Lipofectamine 3000 Transfection Reagent (Thermo Fisher Scientific, USA) according to manufacturer’s manual. In brief, 7.5 × 10^4^ KF-Tx cells were seeded in 24-well plates and allowed to attach. After 24 h, transfection complex including mimic of any of the three assessed miRNAs or scrambled RNA was mixed with cells to final RNA concentration of 5 nM. Cells were collected after 48 h and 750 cells were seeded in 6-well plates and treated the next day with DMSO, 5 nM, 10 nM, 20 nM, 40 nM, and 80 nM PTX followed by incubation for 8 days to allow colonies formation. Colonies were fixed with 70% methanol for 20 min then stained with 0.5% Giemsa stain (Sigma-Aldrich, USA) in 70% methanol for 30 min. The number of colonies, each colony more than 50 cells, was counted using a microscope. The surviving fraction was calculated as the ratio of the plating efficiency of the miRNA-transfected cells to that of scrambled RNA-transfected cells. In order to assess the effect of miRNAs’ mimics on cell viability, KF-Tx or A2780, 2.5 × 10^5^, cells were seeded in 6-well plates and left to attach for 24 h. Then, cells were transfected either with scrambled RNA, miRNA-204-3p, miRNA-3652 or miRNA-4286 mimics. After 48 h, cells were collected and seeded in 96-well plate (5 × 10^3^ cell/well). The next day, cells were treated with PTX with doses ranging from 0.030 to 4 µM for KF-Tx cells and from 0.016 to 1 µM for A2780-Tx cells. After 72 h, cell proliferation was assessed using the Cell Proliferation Kit I (MTT) (Sigma, USA) according to manufacturer’s instructions.

### Western blot

Cell lysates were prepared by lyzing cells in RIPA buffer (10 mM Tris (pH 7.4), 150 mM NaCl, 1% Triton X100, 1% Na.deoxycholate), supplemented with protease inhibitors cocktail (Sigma). Protein concentration of whole cell lysates was determined by BSA assay using the BSA kit (Pierce), then equal protein amounts were heated to 95 °C for 5 min with sodium dodecyl sulfate (SDS) sample buffer (25 ml glycerol, 31.2 ml Tris buffer, 7.5 ml SDS, a dash of bromophenol blue/100 ml) and run on 12% SDS polyacrylamide gel electrophoresis (SDS-PAGE). Protein samples were then blotted onto PVDF membranes (Immobolin P, Watford, UK). The membranes were incubated in blocking solution (5% non-fat milk in PBS) for 1 h then in primary antibody (anti-human AKT2 mAb (at dilution of 20:1000) or GAPDH mAb (at dilution of 20:1000) overnight. After 3 × 10 min washes in TBS (0.1% Tween-20 in PBS) the membrane was incubated for 1 h at room temperature with horseradish-peroxidase-linked IgG (1:2000 dilution in T-TBS) followed by three washes (10 min each) with TBS. Signals on membrane were developed using ECL reagent (Amersham, CA, USA) and then was imaged with Chemidoc system (BioRad, USA).

### Annexin V staining

After treatment, cells were harvested and washed in cold PBS. Then, cells were stained directly with PI at final concentration of 10 μg/ml and 2% Annexin V Flous (BioRad) in incubation buffer (10 mM HEPES/NaOH, pH 7.4, 140 mM NaCl, 5 mM CaCl2) for 10 min. Cells were then acquired with the FACS system (Perkin Elmer) after setting the instrument with controls (nontreated stained cells) after two washes in PBS. In this experiment, both cells with early apoptotic signals, stained with annexin V, and cells with late death signals, stained with PI, are all considered as apoptotic cells. Apoptotic cells were analyzed using the CellExpert software (Perkin Elmer).

### Statistical analysis

Statistical analysis was performed using Minitab Release (Ver.12). Data were subjected to one-way analysis of variance, ANOVA, followed by comparison using student *t* test to evaluate the difference between means. Differences between means were considered significant if *p*-values < 0.05 which is indicated in the figures by (*), highly significant if *p*-values < 0.01 which is indicated by (**) and very highly significant if *p*-values < 0.001 which is indicated by (***).

## Supplementary information

Supplementary Figures.
