# Supplementary material for: Integrative microRNA and gene expression analysis identifies new epigenetically regulated microRNAs mediating taxane resistance in ovarian cancer
Source: Sci Rep. 2021 Jan 12;11:562. doi: 10.1038/s41598-020-78596-5 (PMC7804410; doi:10.1038/s41598-020-78596-5)
Supplement: Supplementary file 1 — Supplementary Figures. [file 41598_2020_78596_MOESM1_ESM.pdf]

Integrative microRNA and gene expression analysis identifies new epigenetically regulated microRNAs mediating taxane resistance in ovarian cancer

Mohamed K. Hassan, Amr Adel Waly, Waheba El-Sayed, Sarah Keshk, Walaa Ramadan Allam and Sherif F. El-khamisy

Supplementary figure (S.1)

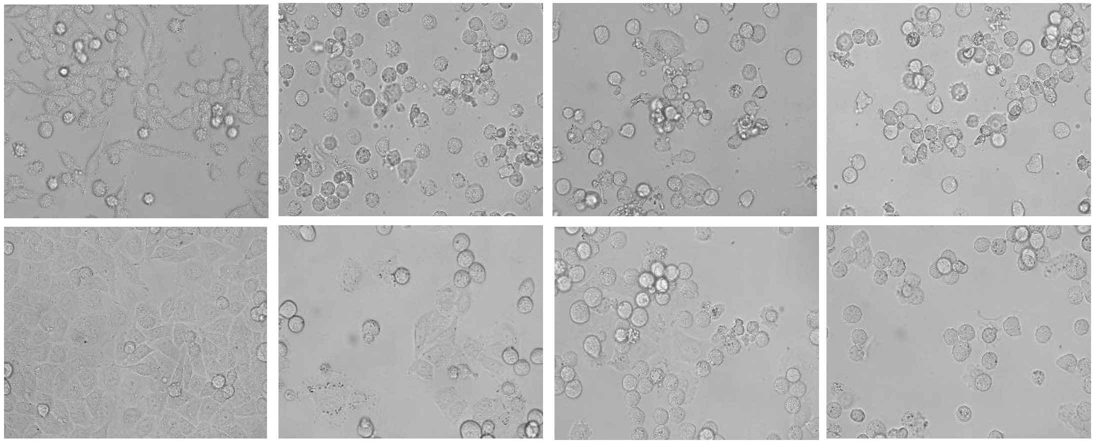

S.1. Phase contrast photos of KF and KF-Tx validating the differential response to PTX . Two cellular clones were cultured For 24h then challenged with indicated concentration of PTX, then photos were captured with Leica microscope.

Supplementary figure (S.2)

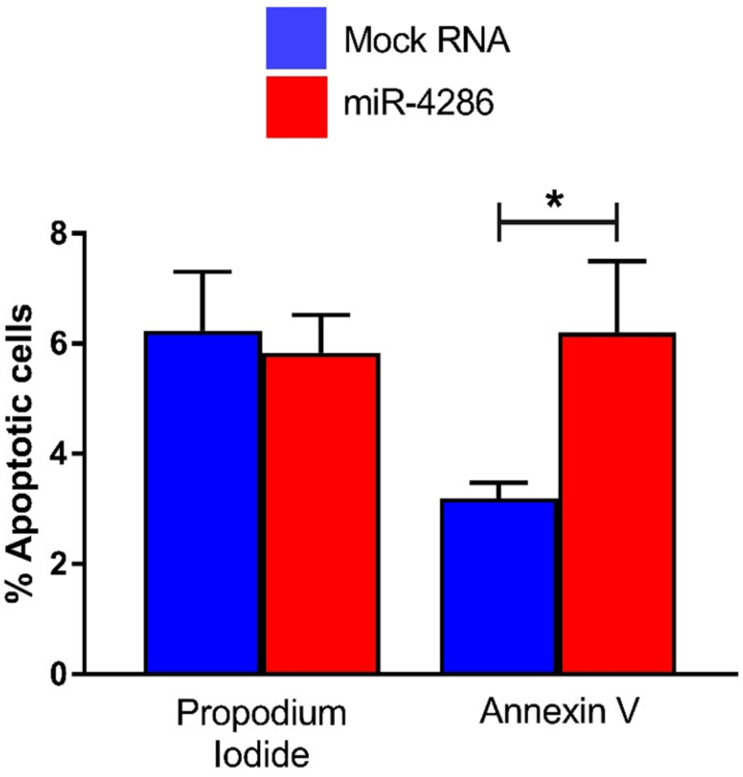

S.2. Average percentage of annexin V stained cells from KF-Tx cells before and after miR-4286 mimic transfection and challenged with PTX (200nM). Cells were doubly stained by PI and annexin V and acquisition was performed by flow cytometer (BD)
